# Land Cover Changes Utilising Landsat Satellite Imageries for the Kumasi Metropolis and Its Adjoining Municipalities in Ghana (1986–2022)

**DOI:** 10.3390/s23052644

**Published:** 2023-02-28

**Authors:** Bernard Fosu Frimpong, Addo Koranteng, Thomas Atta-Darkwa, Opoku Fosu Junior, Tomasz Zawiła-Niedźwiecki

**Affiliations:** 1 Department of Hydrology, Brandenburg University of Technology, Platz der Deutschen Einheit 1, 03046 Cottbus, Germany; 2 Institute of Research, Innovation and Development, Kumasi Technical University, Kumasi P.O. Box 854, Ghana; 3 Department of Agricultural and Bioresources Engineering, University of Energy and Natural Resources, Sunyani P.O. Box 214, Ghana; 4 Institute of Geodesy and Geoinformation Science, Straße des 17. Juni 135, 10623 Berlin, Germany; 5 Coordination Centre for Environmental Projects, Bitwy Warszawskiej 1920 r. 3, 02-362 Warszawa, Poland

**Keywords:** urbanisation, forest loss, land use land cover, support vector machine, correlation, deforestation

## Abstract

Forest loss, unbridled urbanisation, and the loss of arable lands have become contentious issues for the sustainable management of land. Landsat satellite images for 1986, 2003, 2013, and 2022, covering the Kumasi Metropolitan Assembly and its adjoining municipalities, were used to analyse the Land Use Land Cover (LULC) changes. The machine learning algorithm, Support Vector Machine (SVM), was used for the satellite image classification that led to the generation of the LULC maps. The Normalised Difference Vegetation Index (NDVI) and Normalised Difference Built-up Index (NDBI) were analysed to assess the correlations between the indices. The image overlays of the forest and urban extents and the calculation of the annual deforestation rates were evaluated. The study revealed decreasing trends in forestlands, increased urban/built-up areas (similar to the image overlays), and a decline in agricultural lands. However, there was a negative relationship between the NDVI and NDBI. The results corroborate the pressing need for the assessment of LULC utilising satellite sensors. This paper contributes to the existing outlines for evolving land design for the promotion of sustainable land use.

## 1. Introduction

Anthropogenic activities occurring around the world are considered to be the main cause of alterations in Land Use Land Cover (LULC) [1,2]. LULC is a multifaceted sequence of changes prompted by the interaction of human-caused activities and the natural environment impacting the local environment, accumulating into global environmental changes, and impacting sustainable development [3]. Forestlands and agricultural lands have been lost due to human settlements’ expansion [4,5,6,7]. The loss of biodiversity, soil degradation, and soil erosion are other results of urbanisation [8,9]. The ramifications of the LULC changes are severe, as they directly affect the quality of human lives and lead to climate change, environmental changes, and distortions of the ecological functions in biological entities and abiotic components locally, regionally, and globally [10,11].

Urbanisation refers to the development of urban/built-up areas for the provision of houses, industries, and other infrastructure, such as transportation networks and other social amenities that support the existence of humans [12,13,14]. Urbanisation is a common phenomenon globally, but its intensity and dynamism in developing countries because of rapid population growth and economic growth need thorough investigation [15,16]. Several studies have revealed the escalation in the world’s urban population [17,18]. The urban population is estimated to rise to 68% from its present estimate of 55% [19]; to contain this development in urban population, cities are expanding beyond their urban frontiers into peri-urban areas, which exerts pressure on the other LULC types such as forestlands, wetlands, vegetation, and agricultural lands.

Land is a valuable natural resource containing both biotic and abiotic components and has been subjected to anthropogenic developmental activities [20,21,22]. An investigation of the LULC pattern of an area reflects the historical and contemporary status of the resource conditions and their exploitation [23,24]. Consequently, it is imperative to comprehend these dynamics to be able to analyse how they impact human society now and in the foreseeable future [25,26]. The assessment of the trends in the LULC dynamics leads to the identification of the key driving forces behind these trends, as well as their ecological and socioeconomic effects [27]. 

Satellite imagery provides an innovative resource for quantitative and qualitative data that ensures the study of the history, present, and future of the world’s land, atmosphere, and oceans [28,29]. Geospatial technologies such as remote sensing (RS) and geographic information system (GIS) applications offer relatively inexpensive but efficient expertise and have exceptional abilities to display the expanse of the earth to obtain information on the earth [30]. RS and GIS technologies provide efficient means for investigating the LULC dynamics including mapping, monitoring, and managing the environment [31,32]. Investigating the LULC changes through geospatial applications is regarded as vital for researchers, conservationists, engineers, economic experts, politicians, and individuals with an interest in the sustainability of the earth’s natural resources [32,33]. 

The initial studies in Ghana relied on the usage of traditional classifiers for the classification of satellite images especially the Maximum Likelihood Classifier (MLC) [34,35,36,37,38,39,40,41]. The works of [7,42] used the state-of-the-art machine learning algorithm Support Vector Machine; however, little or no attention has given to the use of Support Vector Machine, another state-of-the-art classifier for satellite image classification in Ghana. Moreover, most of the LULC studies in Ghana have concentrated on the main cities of Accra [38], Kumasi [7], and Sekondi-Takoradi [43]. 

In this study, historical and current LULC mapping of the Kumasi Metropolis and adjoining municipalities (Atwima Nwabiagya and Asokore Mampong) of Ghana was conducted. This study evaluated the LULC change for the period 1986–2022. We aim to advance an understanding of the scope and magnitude of the forest loss, the unbridled urbanisation, and other forms of LULC change. In this research, the Support Vector Machines algorithm was used to classify the satellite images from Kumasi, and two adjoining municipalities (Atwima Nwabiagya and Asokore Mampong) were added to provide a broader understanding of the changes in a major city and its adjoining municipalities. 

## 2. Materials and Methods

### 2.1. Study Area

**Figure 1:**
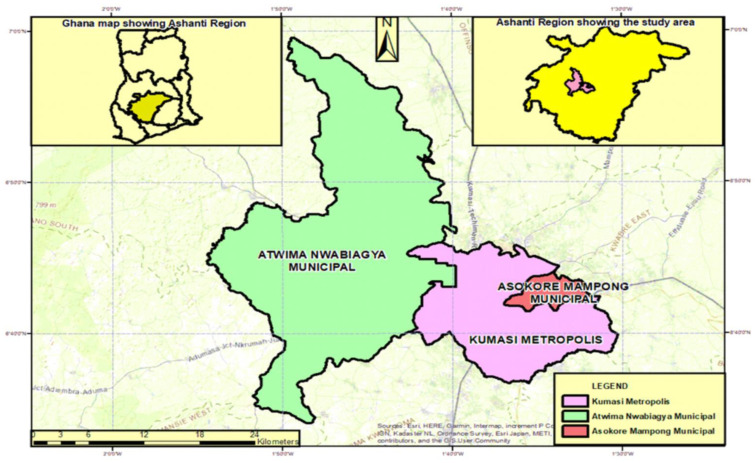
Study Area comprising the Kumasi Metropolitan Assembly, Asokore Mampong Municipality, and Atwima Nwabiagya Municipality.

The Ashanti Region is Ghana’s most populous region with longitude 1°58′ W and 1°11′ W and latitude 6°22′ N and 7°11′ N [44]. This region houses the Kumasi Metropolitan Assembly and the Asokore Mampong and Atwima Nwabiagya Municipalities, which constitute this study (Figure 1). The climate has a constant temperature all year round; it is characterised by both wet and dry seasons, with a mean rainfall measuring 1400 mm annually [44]. 

### 2.2. Data and Software 

Based on the availability and suitability, Landsat images for 1986, 2003, 2013, and 2022 were obtained from the Earth Explorer website of the United States Geological Survey (USGS) (Table 1). Open Street Map (OSM), World Topographic Map (WTM), and World Street Map (WSM) were the ancillary datasets projected to the World Geodetic System (WGS) 84 used in the study. The path and row were 194 and 055 for the downloaded satellite images. ArcGIS software was utilised for this research.

**Figure 2:**
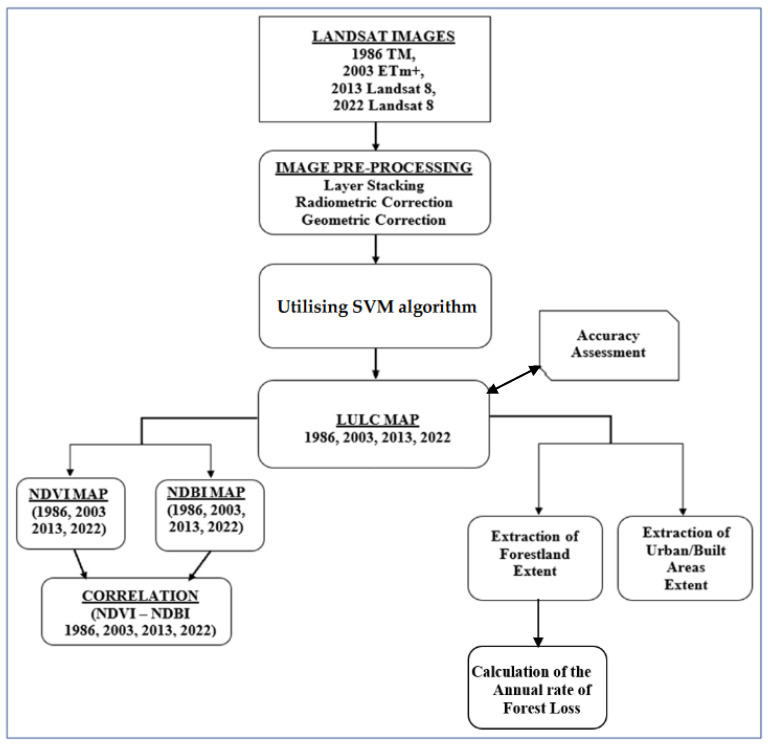
Flowchart of the methodology utilised for the study.

The flowchart shows the steps of the workflow described in this paper (Figure 2).

### 2.3. Image Processing and Classification

Image preprocessing techniques, such as layer stacking (the technique of merging multiple satellite images into a single image), geometric correction (digitally modifying satellite images so the projection of the image matched the particular projection surface), and radiometric correction (processing the satellite images to improve the accuracy of the brightness values), were the initial steps executed in the ArcMap 10.8 software. The Nearest Neighbour Algorithm was used to resample the satellite images. Compared to the other resampling techniques, this algorithm conserves the original values of the downloaded images [45,46]. WGS 84 Universal Transverse Mercator (UTM) Zone 30N and Ghana Datum Office were the projections used. The individual bands were stacked to obtain a composite band, and the area of study was delineated for each year. 

Based on the authors’ local knowledge of the study area and the literature [7,47], four LULC types, urban/built-up areas, agricultural, forestlands, and waterbodies (Table 2), were chosen for the image classification. The Support Vector Machine (SVM) algorithm was employed to allocate pixels to their classes, since the SVM is not limited by statistical assumptions.

### 2.4. Support Vector Machine Classifier

SVM is a supervised nonparametric statistical algorithm that makes no assumption about the distribution of the data [48]. This algorithm uses a hyperplane for the separation into the various LULC classes [49]. SVMs are linear binary classifiers; thus, the technique assigns a class to a test sample, which is classified from the available LULC classes. The hyperplane utilises the decision boundary to segregate the data [50]. In remote sensing, the pixel is the data sample to be utilised. Each pixel is represented as a pattern vector. It is vital to note that, most of the time, not all the available training sets are utilised to describe and specify the separation using the hyperplane [48]. The SVM assumes the pixels are linearly separable in the input space and are separated by utilising the maximum margin of the hyperplane [50]. The segmented attributes used were the colour and the mean in the training of the support vector machine, whilst the maximum number of samples per class was set to fifty. The input training sample features were chosen from the satellite images [51]. The radial basis function was the adopted kernel, as it is the most widely utilised for satellite image classification [52,53,54]. The key parameters needed by the RBF were the penalty value (C) and gamma (ꝩ). The optimal search for the best values for ‘C’ and ‘ꝩ’ was set at the following values: penalty value (C) = 1, gamma = scale (1/(n_features*X.var())), where n_features represented the number of features, X was the pixels’ values (reflectance in the mxn dimension (matrix)), “.” was the dot product, var() is the variance, and kernel = RBF. The best value of ‘C’ was determined by trying out a range of values from 1 to 100. The optimal value of ‘C’ was 50, whilst the other parameters were set to the default.

**Figure 3:**
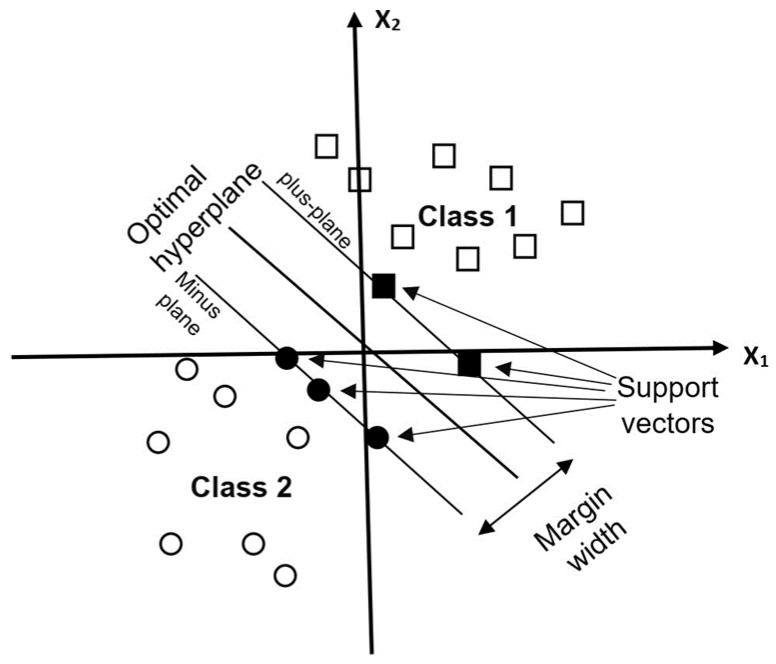
An optimal hyperplane for separating different classes.

The SVMs have the merit of handling a small number of training datasets and producing a higher accuracy after the classification [48]. Figure 3 illustrates the simplest scenario for the classification process utilising the hyperplane.

### 2.5. Accuracy Assessment

An accuracy assessment was performed for all the classified images. The assessment report was produced as confusion matrices. Two hundred ground control points were used, with 50 points chosen utilising the equalised sampling technique for each class (urban/built-up, agricultural lands, forestlands, and waterbodies). The evaluation and validation were conducted using the control points selected from the satellite images. The ancillary datasets (World Topographic Map, Open Street Map, Google Earth Historical Imagery, and World Street Map) were used as reference maps for the identification of the various LULC classes. 

### 2.6. NDVI and NDBI Analysis

#### 2.6.1. Normalised Difference Vegetation Index

The Normalised Difference Vegetation Index is the most commonly used remotely detected vegetation index [55]. 

The near infrared (NIR) and the red (R) bands were used for the computation. The NDVI was calculated as:

(1) $$\mathrm{NDVI}=\frac{\mathrm{NIR}-R}{\begin{array}{c} \;\mathrm{NIR}+R \end{array}}$$

**Figure 4:**
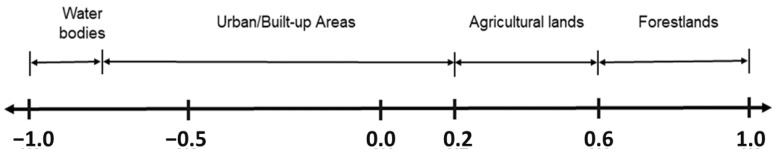
Diagrammatic representation of the LULC classes and their associated values.

The NDVI values vary from −1 to +1. Waterbodies usually record values close to −1. The urban/built-up areas produce very low NDVI values (0.10 or less), whilst agricultural lands have moderate values (0.20 to 0.59). NDVI values from 0.6 to 1.0 are equated to dense vegetation (i.e., forestlands) [56] (Figure 4).

#### 2.6.2. Normalised Difference Built-Up Index

The NDVI is regarded as one of the spectral indices used to extract urban/built-up areas. It is a good indicator of urban/built-up features due to its high reflectivity in the SWIR band, rather than in the NIR band [57]. 

Thus, it was computed utilising the equation:

(2) $$\;\mathrm{NDBI}=\frac{\mathrm{SWIR}-\mathrm{NIR}}{\begin{array}{c} \mathrm{SWIR}+\mathrm{NIR} \end{array}}$$

The NDBI values span from −1 to +1. The nearer the value to +1, the higher the value for urban/built-up areas and vice versa [57].

#### 2.6.3. NDVI–NDBI Correlation

The relationship between the NDVI and the NDBI was determined by creating points in the study area using the fishnet tool in ArcMap. In total, 980 points were created. The ArcMap tool ‘Extract values to points’ was used to extract the NDVI and NDBI values for the selected years of study.

### 2.7. Rate of Deforestation 

The calculated annual rate of deforestation used in this research was adopted from the works of [58,59]. This rate provides the average speed at which deforestation occurs, and it is stated in unit area per year.

(3) $$R=\frac{A_{1}-A_{2}}{\begin{array}{c} t_{2}-t_{1} \end{array}}$$

where R = rate of deforestation

A_1_ = initial value for forestlands in hectares

A_2_ = second yearly value of forestlands in hectares

t_2_ = second year 

t_1_ = initial year 

## 3. Results

### 3.1. Distribution Patterns and Trends of the LULC Classes

**Figure 5:**
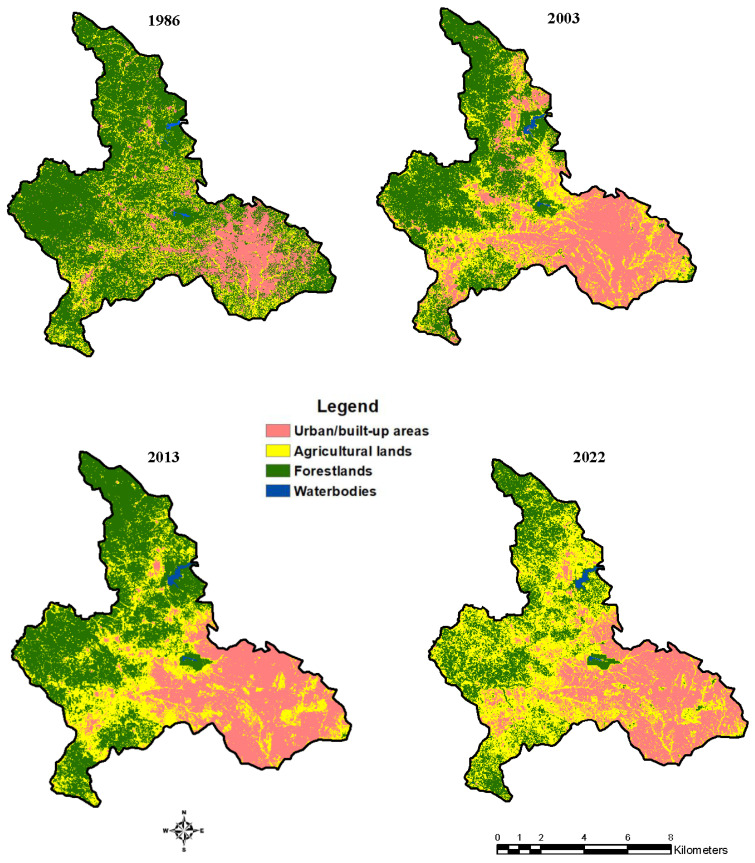
Land use land cover maps (1986, 2003, 2013, and 2022).

The study area was categorised into four classes: urban/built-up areas, agricultural lands, forestlands, and waterbodies. The urban/built-up areas were concentrated in the southeastern portions of the study area. However, the classified image of 2003 revealed that the built-up areas were gradually spreading towards the northern and southern parts of the study area. The classified images of 2013 and 2022 revealed that the southeastern parts were almost all urban/built-up areas. The agricultural lands increased from 1986 to 2003, decreased in 2013, and increased in the final year of the study. The forestlands decreased significantly in the year 2003, increased steadily after 2003, but further declined in the last year. The waterbodies remained constant throughout the study period (Figure 5).

The urban/built-up areas that covered approximately 15% in 1986 increased to 30.68% in 2022. Agricultural lands increased from 19.47% in 1986 to 42.71% in 2022. Forestlands that covered 65.15% in 1986 were reduced to 26.17% in 2022. On the other hand, the smallest changes in the LULC were observed for the waterbodies, as a slight increase was observed from 0.33% (1986) to 0.44% (2022) (Table 3).

### 3.2. Land Use Land Cover Changes

**Figure 6:**
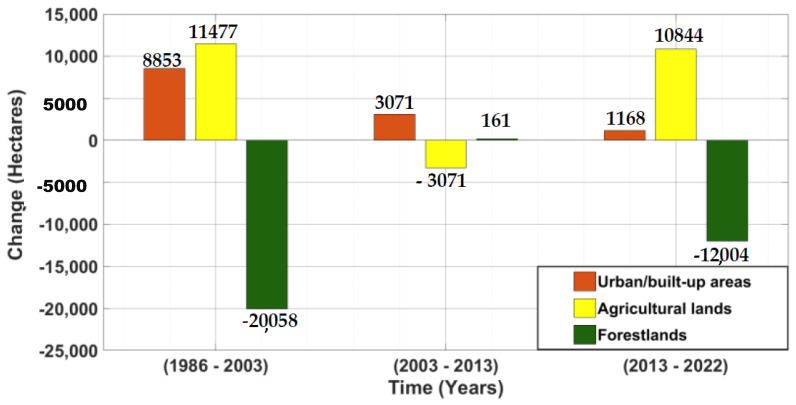
LULC change in hectares.

The land use land cover changes revealed increasing trends for the urban/built-up areas throughout the study period, although there was a decline in the amount of gained hectares from 1986 to 2022. From 1986 to 2003, there was an increase in agricultural lands, with a decline from 2003 to 2013. However, there was a rise from 2013 to 2022. On the other hand, the forestlands decreased the most from 1986 to 2003. They increased slightly from 2003 to 2013 and further decreased from 2013 to 2022 (Figure 6).

### 3.3. Accuracy Assessment

The last stage of the satellite image classification using the support vector machine algorithm recorded the kappa statistics for 1986, 2003, 2013, and 2022 as 95%, 97%, 97%, and 98%, respectively. The user accuracy ranged from 96% to 100%. However, the producer accuracies were from 92% to 100% (Appendix A).

### 3.4. NDVI and NDBI Analysis

**Figure 7:**
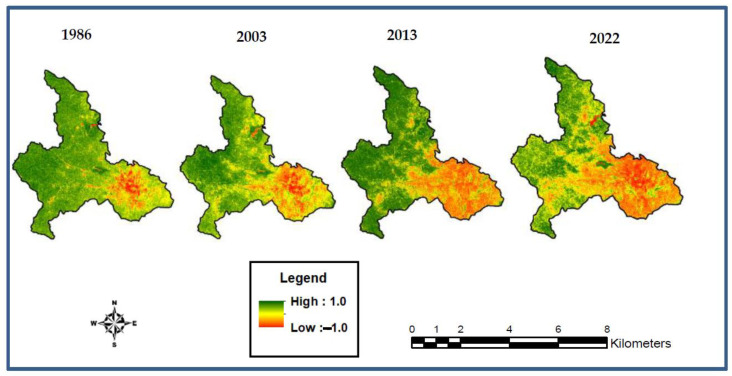
NDVI maps of the study area.

The range of values was from −1 to 1. Matching the NDVI maps to the LULC maps, it was evident that the lowest values were produced in the water environment. The urban/built-up areas also yielded lower values. The agricultural lands produced higher NDVI values. However, the lands classified as forests yielded the highest values for the NDVI. The pattern was uniform for the NDVI maps of 1986, 2003, 2013, and 2022 (Figure 7). 

**Figure 8:**
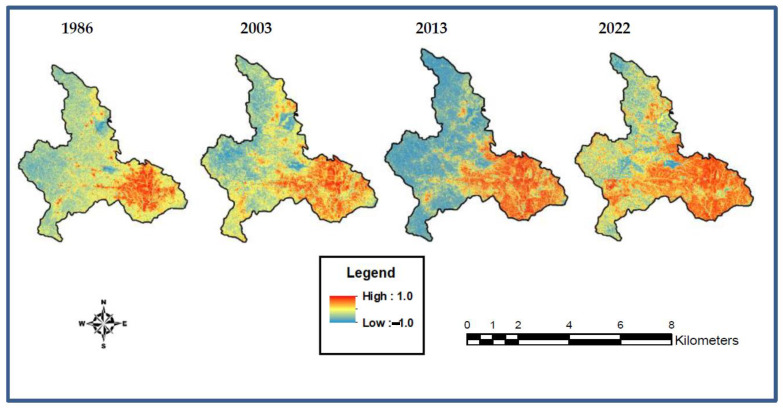
NDBI maps of the study area.

The NDBI is sensitive to urban/built-up areas. The values ranged from −1 to 1. Compared to the LULC maps, the lowest values were recorded in the water environment, followed by the agricultural lands and forestlands. The highest values were recorded in the urban/built-up areas (Figure 8). 

#### 3.4.1. Forest Extent

**Figure 9:**
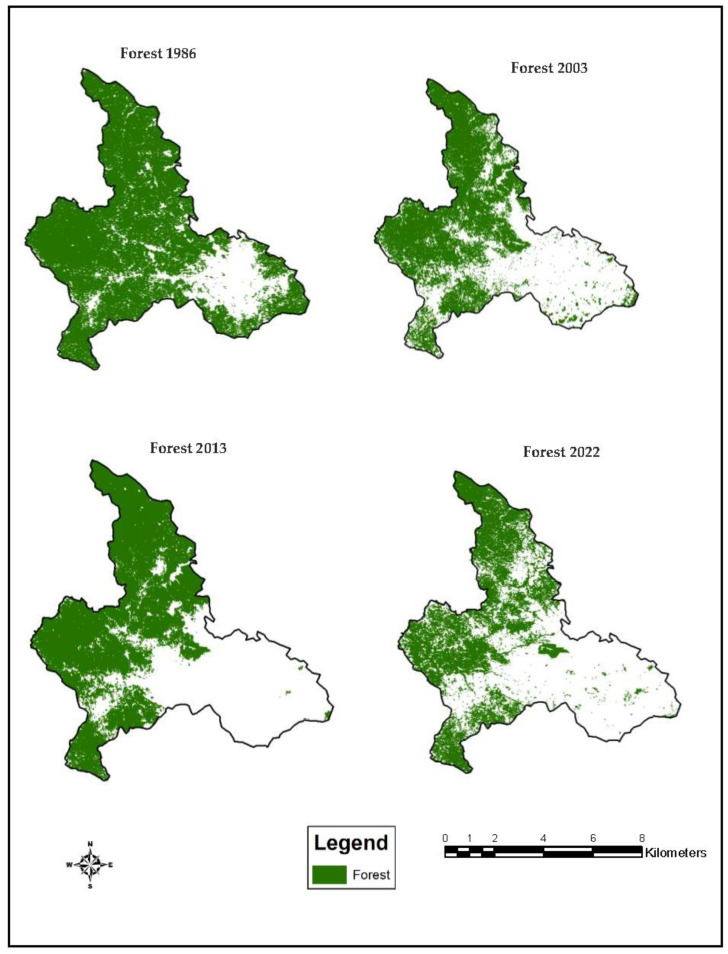
Forest extent maps.

The forestlands were determined using overlays of the satellite images. The forest was the dominant class in 1986 (53,327 ha) and covered most of the portions in the study. However, in the forest extent of 2003, the forestlands were mainly located in the southeastern parts, i.e., 33,269.43 ha. Forestlands were lost towards the northern and southern parts of the study area. Additionally, the forestland declined and was almost lost at the central, eastern, and southern peripheries in 2013, where the forestlands were 33,430 ha. The forest extent of 2022 revealed some traces of forestlands at the central, eastern, and southern portions of the study area. The entire amount of forestland was 21, 426 ha with lost traces and clusters of forestlands in the year 2022 (Figure 9). 

#### 3.4.2. Urban Extent

**Figure 10:**
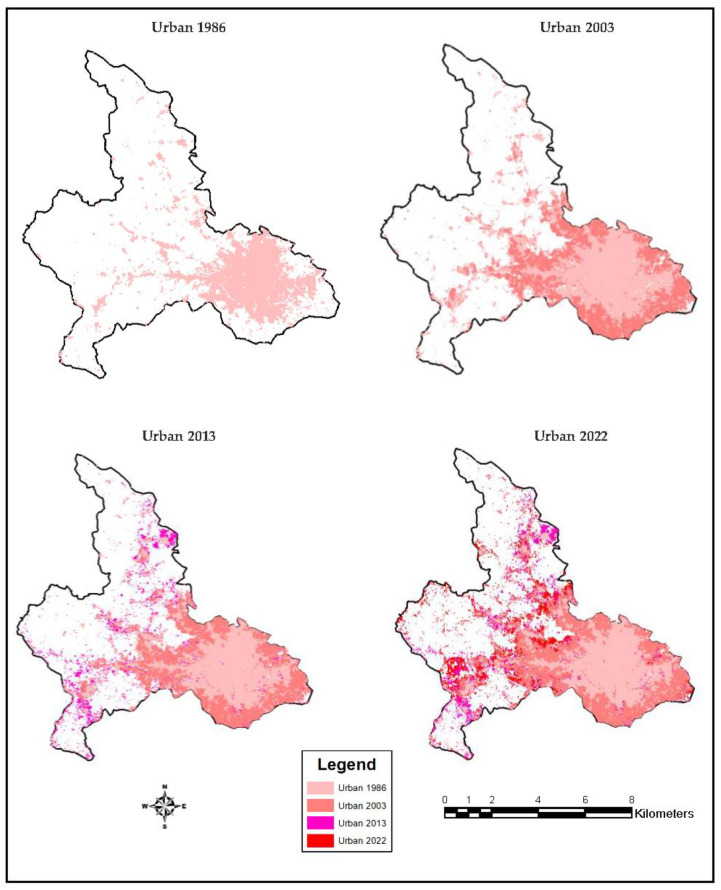
Urban extent maps.

The trends in urban expansion were extracted from the classified satellite images. The urban/built-up areas were dominantly observed in the southeastern portions in 1986 (12,318 ha). There were traces of urban/built-up areas towards the northern and eastern portions. However, it was found that the peripheries of the urban/built-up areas that belonged to other LULC classes changed into urban/built-up areas in 2003 with the total urban/built-up areas recording 20,871 ha. The urban/built-up areas in 2013 (23,942 ha) revealed an increasing trend to the detriment of the other LULC classes. Urban/built-up areas again increased in the last year of the study (25,110 ha) (Figure 10). 

#### 3.4.3. NDVI–NDBI Correlation

The correlation analysis documented the relationship between the Normalised Difference Vegetation Index (NDVI) and the Normalised Difference Built-up Index (NDBI). The correlation ranged from −0.98 to −0.89 throughout the study years. The range of values indicated a strong inverse relationship between the NDVI and the NDBI. However, the strongest and weakest correlations were recorded for the years 2013 and 1986, respectively (Table 4). 

### 3.5. Annual Rate of Deforestation

The annual rate of deforestation evaluates the gains/losses in the forestlands. It was found that from 1986 to 2003, 1180 hectares of forestlands were lost annually. In contrast, from 2003 to 2013, there was an annual increase of 16 hectares per year. Finally, between 2013 and 2022, 1334 hectares of forestlands were lost to other LULC categories annually, and this represented the highest amount of lost forestlands (Table 5). 

## 4. Discussion

### 4.1. Satellite Image Classification and Accuracy Assessment 

The support vector machine algorithm was utilised for the classification of satellite images. The efficacy of the SVM algorithm used in the works of [60,61,62] was higher compared to the traditional classifiers utilised for satellite image classification. 

SVM achieved a higher level of classification accuracy than either the Machine Learning (ML) or the Artificial Neural Networks (ANN) classifier in [63]. The research of [64] tested nine different supervised classification techniques (neural network, spectral angle mapper, maximum likelihood, SVM, Mahalanobis distance, binary code, minimum distance, spectral information divergence, and parallelepiped) for LULC mapping in the province of Mazandaran, Iran. The results indicated the Support Vector Machine (SVM) classifier had the best accuracy compared to the other classifiers. SVM outperformed other image classification algorithms in terms of accuracy in [53,63,65,66]. The results of this study support the findings of [62,67], which found that SVM had a higher accuracy than other image classification algorithms such as ANN, minimum distance, and other machine learning algorithms. The work of [68] also found that SVM was the most efficient algorithm for most applications and outperformed several classifiers, including random forest (RF), neural networks (NN), and decision trees (DT) in direct comparison. SVM performed better than the maximum likelihood classifiers and parallelepiped classification techniques in [69]. In terms of classification algorithms, SVM achieved the highest accuracy, followed by the neural network techniques. The random forest classifier performed considerably better than the traditional decision tree classifier. The SVM method of classification technique provided a better result than MLC and ANN in [70,71,72]. 

The SVM was a fast and accurate classifier, and it is highly recommended for studies on LULC changes [62]. The kappa statistic and producer and user accuracies yielded satisfactory results that were higher than previous research that utilised the traditional parameters in other parts of Ghana including the works of [34,40]. It was obvious that the machine learning algorithms, for instance, support vector machine, performed better in terms of the satellite image classification than the traditional classifiers.

### 4.2. Land Use Land Cover Changes

The LULC maps revealed that most urban/built-up areas were located in the south-eastern parts of the study area. The depletion of agricultural lands might be attributed to the rise in the demand for land for commercial centres and residential areas. The analysis of the LULC revealed that forestlands have been subjected to intense pressure due to anthropogenic activities [27]. The conversion of forestlands and agricultural lands into urban/built-up areas is considered positive in the Ghanaian setting [34,37,40,73]. The rise in population is a major factor that may have triggered the expansion of the built-up areas, and it supported the outcome of the works of [35,74,75]. The population living in peri-urban areas tends to rely on forest resources for sustenance [76]. It was found that agricultural lands were the first to be converted to built-up areas [27]. This is because people buy lands in peri-urban areas, where the land for the development of urban/built-up areas has relatively lower prices, and these areas were formerly agricultural lands [77]. Hence, the urban/built-up areas grow outwards from the already existing urban/built-up centres.

In Ghana, house ownership serves as a higher indicator of one’s social status in society [78]. Land ownership is vital for attaining pride, which is an important part of the traditions and culture of Ghanaians [79]. Marginal gains or losses were observed for waterbodies, which implied that the immediate borders of the waterbodies have been slightly affected during the study period. The high rate of deforestation especially from 2013 to 2022 could be attributed to the high demand for fuelwood, products from the forest for settlement purposes, and infrastructure projects. The increase in the forestlands from 2003 to 2013 could be attributed to the afforestation activities that were embarked upon in the study area. The increased urban/built-up areas could be ascribed to the surging population and unbridled urbanisation.

### 4.3. NDVI–NDBI Correlation

The correlation between the NDVI and NDBI could be described as a strong inverse relationship. This meant that points with higher values for NDBI had lower values for NDVI and vice versa. This implied that the urban/built-up areas yielded lower values for NDVI. This outcome collaborated with the findings of [80] that there was a negative relationship between the NDVI and NDBI.

### 4.4. Urban and Forest Extent

The urban extent increased throughout the study years. This could be attributed to the desire for developmental projects in the study area. The analysis revealed a strong rise in the acquisition of land for building purposes. The urban/built-up areas increased to the detriment of other LULC types especially forestlands and agricultural lands. This assertion was supported by the outcome of the earlier works of [34,47]. 

The forest cover was the most depleted LULC type during the study period (Table 4). This may be attributed to the policies that motivated people to log trees as revealed in the Structural Adjustment Programme (SAP) that led to the establishment of timber logging firms and helped to raise foreign exchange to service Ghana’s debt [81]. There was a deliberate reduction in the number of forest guards in the various forest reserves. The SAP led to an increased number of civil servants becoming timber loggers [82]. Thus, it was accepted in the context of Ghanaian society that the forest conversions were legal, intentional, and essential for national development [83]. However, from 2003 to 2013, the increase in the annual rate of forestlands was attributed to the establishment of plantations across the country by the Forestry Commission of Ghana [84]. Nonetheless, there was an extensive loss of forestlands from 2013 to 2022 [85]. This may be attributed to the fact that urbanisation was on the rise during that period [7]. In addition, the western and northern regions have witnessed a lot of illegal mining activities [86]. This has led to the lands being exposed to soil degradation and pollution [87]. 

## 5. Conclusions

This study illustrated the efficiency of satellite images for insights into monitoring and evaluating the spatiotemporal alterations in LULC in the Kumasi Metropolis and Atwima Nwabiagya and Asokore Mampong Municipalities in Ghana from 1986 to 2022. The classification was performed utilising the SVM algorithm. The study extracted the forest and urban/built-up extents for the study period. The evaluation of the NDVI, NDBI, and the relationship between the two was also completed. 

The study revealed an increasing trend in the urban/built-up areas throughout the study period. The forestlands declined from 1986 to 2022. The research revealed that the SVM algorithm was a better classifier compared to other classification algorithms. The results also revealed that areas classified as urban/built-up areas had higher values for the NDBI and lower values for the NDVI. The reverse was true for the agricultural and forestlands (low NDBI and high NDVI). The relationship between the NDVI and NDBI revealed a negative correlation. The study concluded that the increasing urban/built-up areas had a substantial impact on greenery (agricultural lands and forestlands). The RS indices (NDVI/NDBI) incorporated in this research provided deeper insights into the natural environment of the study area.

The key limitation of the study was the inability to obtain at least a 10-year interval from 1986 to 2022 due to the satellite imagery challenges, and very high-resolution images were not possible for such a retrospective study. Nonetheless, the technique utilised in this study was considered straightforward. It is recommended that Landsat images of the wet season may be utilised and compared for satellite image classification.

This study proposes that land usage in the study area for developmental projects especially for residential purposes should be reviewed properly by the relevant authorities to avoid the destruction of agricultural and forestlands. Land use policies should be enforced in the study area to curtail the rampant destruction of the greenery in the study area. 

This research highlighted the changes and trends in the various LULC classes utilising the machine learning algorithm, SVM, in terms of the extracted forests, urban/built-up areas, and the RS indices (NDVI and NDBI). This study contributes to the current framework for developing land planning for the promotion of the sustainable use of forestlands and incorporating urban/built-up areas into other LULC types and proved the efficacy of satellite imagery for monitoring earth resources.

## Figures and Tables

**Table 1:** Raster data utilised in this study.

Satellite	Acquisition Date	Number of Bands	Resolution (m)	Percentage Cloud Cover
**Landsat Series**				
Thematic Mapper (TM)—5	11.01.1986	6	30	1.00
Enhanced Thematic Mapper plus (ETM+)—7	19.02.2003	7	30	3.00
Operational Land Imager (OLI)—8	23.12.2013	9	30	0.86
Operational Land Imager (OLI)—8	03.03.2022	9	30	0.92

**Table 2:** The land use land cover classes adopted for the study.

Land Cover Category	Description	
Urban/Built-up	Residential	Commercial
	Industrial	Power and communications facilities
	Highways and transportation	Institutions
	Lands with exposed soil surface	
Agricultural Lands	Cropland	Pasture
	Horticultural areas	
Forestlands	Mixed forestlands: short canopy trees of about 5–10 m high with a thin occurrence of some emergent trees	
Water	Rivers	Streams
	Lakes	Lagoons
	Wetlands	

**Table 3:** Quantification of land use land cover.

LULC CATEGORY	1986		2003		2013		2022	
	(Ha)	(%)	(Ha)	(%)	(Ha)	(%)	(Ha)	(%)
Urban/built-up	12,318.20	15.05	20,871.35	25.50	23,942.62	29.25	25,110.83	30.68
Agricultural lands	15,941.26	19.47	27,418.24	33.49	24,119.72	29.47	34,963.17	42.71
Forestlands	53,327.21	65.15	33,269.43	40.64	33,430.50	40.84	21,426.15	26.17
Waterbodies	271.45	0.33	299.10	0.37	365.27	0.44	357.98	0.44
TOTAL	81,858.12	100.00	81,858.12	100.00	81,858.12	100.00	81,858.12	100

Ha = Hectares.

**Table 4:** NDVI–NDBI Correlation.

Year	Correlation (NDVI/NDBI)
1986	−0.89
2003	−0.91
2013	−0.98
2022	−0.92

**Table 5:** Annual Deforestation Rate.

Period	Annual Rate of Deforestation (Ha per Year)
1986–2003	1180
2003–2013	−16
2013–2022	1334

## Data Availability

Data supporting the findings of this study are available from the corresponding author (BFF) on request.

## Appendix A

**Table A1:** User, Producer, and Kappa statistics for the classified image of 1986.

OID	Class Value	Urban/Built Up	Agriculture	Forestlands	Water	Total	User Accuracy	Kappa
0	Urban/Built-up	50	0	0	0	50	1	0
1	Agriculture	0	48	2	0	50	0.96	0
2	Forestlands	0	1	49	0	50	0.98	0
3	Water	0	0	0	50	50	1	0
4	Total	50	50	50	50	200	0	0
5	Producer Accuracy	0.92	0.96	1	1	0	0.96	0
6	Kappa	0	0	0	0	0	0	**0.95**

**Table A2:** User, Producer, and Kappa statistics for the classified image of 2003.

OID	Class Value	Urban/Built Up	Agriculture	Forestlands	Water	Total	User Accuracy	Kappa
0	Urban/Built-up	0	0	50	0	50	1	0
1	Agriculture	1	49	0	0	50	0.98	0
2	Forestlands	0	1	49	0	50	0.98	0
3	Water	0	0	0	50	50	1	0
4	Total	50	50	50	50	200	0	0
5	Producer Accuracy	0.94	0.98	1	1	0	0.98	0
6	Kappa	0	0	0	0	0	0	**0.97**

**Table A3:** User, Producer, and Kappa statistics for the classified image of 2013.

OID	Class Value	Urban/Built Up	Agriculture	Forestlands	Water	Total	User Accuracy	Kappa
0	Urban/Built-up	50	0	0	0	50	1	0
1	Agriculture	0	49	1	0	50	0.98	0
2	Forestlands	0	1	49	0	50	0.98	0
3	Water	0	0	0	50	50	1	0
4	Total	50	50	50	50	200	0	0
5	Producer Accuracy	0.92	0.96	1	1	0	0.99	0
6	Kappa	0	0	0	0	0	0	**0.97**

**Table A4:** User, Producer, and Kappa statistics for the classified image of 2022.

OID	Class Value	Urban/Built Up	Agriculture	Forestlands	Water	Total	User Accuracy	Kappa
0	Urban/Built-up	50	0	0	0	50	1	0
1	Agriculture	0	48	2	0	50	0.96	0
2	Forestlands	0	0	50	0	50	1	0
3	Water	0	0	0	50	50	1	0
4	Total	50	50	50	50	200	0	0
5	Producer Accuracy	0.92	0.96	1	1	0	0.99	0
6	Kappa	0	0	0	0	0	0	**0.98**
